# Engineered Fenretinide- and Tocilizumab-Releasing Janus Nanoparticles for Site-Directed Immunochemoprevention of Squamous Cell Carcinoma of the Lung

**DOI:** 10.3390/pharmaceutics17111471

**Published:** 2025-11-14

**Authors:** Daren Wang, Albert Chang, Fortune Shea, Yifei He, Richard Spinney, Jonathan D. Whitsett, Joerg Lahann, Susan R. Mallery

**Affiliations:** 1 Division of Oral & Maxillofacial Pathology, College of Dentistry, The Ohio State University, 305 W 12th Ave., Columbus, OH 43210, USA; 2 Biointerfaces Institute, University of Michigan, Ann Arbor, MI 48105, USA; albechan@umich.edu (A.C.); robinyf@umich.edu (Y.H.);; 3 Department of Material Science and Engineering, University of Michigan, Ann Arbor, MI 48105, USA; 4 Department of Chemistry and Biochemistry, The Ohio State University, Columbus, OH 43210, USA; 5 Department of Biomedical Engineering, University of Michigan, Ann Arbor, MI 48105, USA; 6 Department of Chemical Engineering, University of Michigan, Ann Arbor, MI 48105, USA; 7 The Ohio State University Comprehensive Cancer Center, Columbus, OH 43210, USA

**Keywords:** lung cancer, fenretinide, Janus nanoparticles

## Abstract

**Background**: Both clinical and research data support the contribution of IL6-mediated local immunosuppression coupled with IL6-initiated protumorigenic processes, e.g., sustained proliferation and angiogenesis in the development of many cancers, including lung cancer. By virtue of their pharmacologic advantage, controlled release, local delivery formulations can provide immunochemopreventive relevant agent levels at the target site with negligible systemic agent-related effects. Bioavailability is a major challenge with chemopreventive agents. **Methods**: Janus nanoparticles (JNPs), however, are a versatile drug delivery platform that addresses several major cancer preventive challenges including bioavailability and retention of bioactivity, with elimination of potential deleterious effects with systemic administration. Furthermore, JNPs feature two discrete compartments that enable concurrent delivery of two chemically distinct agents with complementary mechanisms of action. **Results**: Our data show that the synthetic vitamin A derivative, fenretinide (4HPR), and the IL6R inhibitor, tocilizumab (TCZ), inhibit pathways integral for the development of lung cancer. Initial molecular modeling and kinase activity assays confirmed that 4HPR serves as a competitive inhibitor for active-site ATP binding of two key IL6 downstream kinases (JAK1, CK2). Concurrent RNA-seq analyses that employed Qiagen Ingenuity Pathway Analysis showed significant inhibition of canonical pathways associated with DNA replication and division in conjunction with significant activation of immunogeneic cell death and TREM 1 signaling pathways and showed the immune-augmenting, cancer-preventive impact of 4HPR-TCZ treatment on gene expression in premalignant lung epithelial cells. Subsequent qRT-PCR analyses corroborated the RNA seq findings and demonstrated 3- to 6-fold increased expression of TREM 1 and immunogenic cell death genes, such as TREM1 and NLRC4 and HSPA6 and DDTT3, respectively. These data collectively guided the development of human serum albumin–chitosan JNPs for the co-delivery of 4HPR and TCZ, respectively. 4HPR-TCZ JNP characterization studies demonstrated high circularities and stability in suspension, as shown by consistency in diameter and minimal changes to the polydispersity index, while confocal microscopy confirmed their biocompartmental nature. Subsequent tertiary chemoprevention in vivo studies that employed a highly aggressive human lung cancer cell line showed that JNPs releasing 4HPR and 4HPR-TCZ significantly reduced tumor volume, as assessed by vital tumor tissue, suppressed proliferation, increased apoptosis, and promoted intratumor vascular instability. **Conclusions**: Collectively, these studies elucidate 4HPR-TCZ in vitro chemopreventive mechanisms of action and demonstrate proof of concept for JNP-4HPR-TCZ in vivo efficacy.

## 1. Introduction

Despite extensive smoking cessation campaigns, advances in surveillance and focused treatments, lung cancer remains the leading cause of cancer-related death worldwide [1]. In 2024, over 234,500 persons in the US received a lung cancer diagnosis, while worldwide lung cancer diagnoses approached 2.5 million [1,2]. The pervasiveness of lung cancer, the associated socio-economic costs of treatment, and the loss and suffering for those affected all combine to create a compelling case for effective, well-tolerated, and convenient lung chemoprevention. Immunochemoprevention entails the use of natural or synthetic substances to enable the hosts’ immune response and prevent cancer development or recurrence. In contrast, chemotherapy is administered following a cancer diagnosis, includes use of toxicity-inducing genotoxic drugs, and is rarely curative.

The majority of lung cancer chemoprevention trials to date have employed systemic administration of repurposed drugs, which resulted in a lack of efficacy, undesirable side effects, and agent inactivation by first-pass metabolism. Via their pharmacologic advantage, local delivery formulations can deliver relevant levels of agents to the treatment site without deleterious systemic drug-related side effects [3,4]. Although chemopreventives have notoriously poor bioavailability, local delivery formulations can also address these challenges. While many local delivery strategies exist, e.g., polymeric implants, standard nanoparticles, our team has found that Janus nanoparticles (JNPs) are particularly well-suited for chemoprevention. JNPs are a versatile platform capable of delivering an array of agents, including siRNA, proteins, and biologics [5,6,7,8]. Furthermore, the two discrete hemispheres (i.e., compartments) in JNPs enable concurrent delivery of two chemically distinct agents with complementary mechanisms of action. Clinical data have confirmed that reliance on single-agent treatment becomes ineffective due to the multiple redundancies in key pathways. To circumvent this issue, JNPs enable concurrent delivery of complementary agents that target unique cancer-promoting pathways, thereby reducing the potential for agent resistance. Furthermore, JNPs can preserve and stabilize complex tertiary structures, which is vital during aerosolization, and enable delivery of bioactive agents with challenging pharmacological profiles, e.g., highly hydrophobic drugs. Another central consideration is that the JNPs must be optimized in accordance with the treatment site, e.g., the capacity to penetrate pulmonary mucus and surfactant while minimizing pulmonary irritation. Furthermore, if particle composition is insufficient to optimize adherence at the target site, JNPs are amenable to surface decorations, e.g., targeting receptors, to augment uptake by progenitor squamous cells of lung squamous cell carcinoma.

Squamous cell carcinoma of the lung (LUSC), which represents approximately 20–30% of non-small cell lung cancers [9], is an excellent starting point for lung cancer immunochemopreventive strategies. LUSC has a clinically recognizable precursor lesion, i.e., a premalignant lung lesion (PML) [10,11], which can be directly visualized by upper airway endoscopy. Endoscopic visualization enables pretreatment biopsies and clinical monitoring. Also, the LUSC “at-risk” cohort is readily identifiable and includes high-grade and/or recurrent-persistent PML, history of smoking, chemical/environmental exposures, e.g., gasoline, diesel, and radon and chronic obstructive pulmonary disease (COPD)/sustained chronic lung inflammation [9]. LUSCs arise in the large, upper airways, which present a short, nearly direct target from the trachea for aerosolized delivery. In addition, the upper airway treatment target site spares the terminal alveoli, which are vital for air exchange and pulmonary function.

Optimal immunochemoprevention targets LUSC enabling mediators, such as elevated intrapulmonary IL6, inappropriately sustained proliferation, and resistance to programmed cell death. Via intracrine, paracrine, and trans-signaling, IL6 activates the JAK-STAT3 signaling hub in both lesional and stromal cells, which stimulates proliferation and angiogenesis and perpetuates local inflammation [12]. IL6 also drives tumor microenvironment immunosuppression through multiple mechanisms, including reduced expression of MHC-II, CD80/86, and IL-12 in dendritic cells, differentiation of dendritic cells to immunosuppressive M2 macrophages [13], and suppression of T-cell-mediated anti-tumor immunity [14]. IL6-initiated JAK1-STAT3 signaling also increases immune-inhibiting PD-L1 expression and augments PD-L1 stabilization via phosphorylation. By virtue of its ability to mask neoantigens expressed during premalignant disease, stabilized PD-L1 provides immune evasion, thus enabling LUSC carcinogenesis [15].

Accordingly, this study focused on strategies to disrupt this proinflammatory, immunosuppressive cascade and disrupt lung cancer tumorigenesis [16,17]. Previously, our team demonstrated that combined treatment with the growth-regulatory, synthetic vitamin A derivative fenretinide (4HPR) and the humanized IL6R receptor-blocking antibody tocilizumab (TCZ) provide complementary cancer-preventive effects [18]. The additive chemopreventive effects of these two agents reflect their respective, complementary mechanisms of action. Specifically, 4HPR regulates cell growth via hTERT inhibition, induction of apoptosis and terminal differentiation [19,20] while concurrently functioning as a small-molecule kinase inhibitor to suppress gratuitous signaling and inhibit directed migration and invasion [18,20,21]. Via competitive interference with IL6 at the IL6R, TCZ reduces the IL6-initiated, pro-inflammatory, immunosuppressive, pro-angiogenic milieu present in PML lesions.

The majority of previous lung cancer prevention studies have relied on systemic agent administration of repurposed drugs, which have resulted in modest to negligible benefits. Accordingly, the objective of this study was to assess the chemopreventive impact of local delivery of two agents with complementary cancer preventive mechanisms of action, i.e., 4HPR and TCZ. Initial investigations confirmed that the kinase inhibitory function of 4HPR extends to key kinases downstream of IL6 (JAK1 and CK2). Concurrent RNA-seq analyses corroborated the immunochemopreventive impact of 4HPR-TCZ treatment on gene expression in premalignant lung cells. These data collectively guided the development of human serum albumin-chitosan JNPs for the co-delivery of 4HPR and TCZ, respectively. Subsequent proof-of-concept in vivo studies that employed a highly aggressive, 100% tumorigenic human LUSC cell line confirm the impact of 4HPR-TCZ JNPs on tumor size, vascular stability, proliferation, and apoptotic indices. To our knowledge, this study is the first to report 4HPR’s competitive inhibition of two kinases (JAK1 and CK2) that are integral for IL6-mediated carcinogenesis and to confirm the in vivo cancer-preventive efficacy of JNP-mediated local delivery of 4HPR and TCZ.

## 2. Materials and Methods

### 2.1. Molecular Modeling to Assess Capacity of 4HPR to Function as a Competitive Inhibitor for the ATP-Binding Sites of the PD-L1 Phosphorylating Kinases, JAK1 and CK2

To investigate the binding interactions and structural differences among Janus kinases (JAK1, JAK2, ad JAK3), and casein kinase 2 (CK2), we employed high-resolution crystal structures retrieved from the Protein Data Bank, https://www.rcsb.org (accessed on 10 July 2024) [22]. For the JNK family, we selected JAK complexed with an ATP-site inhibitor (JAK1 PDB-3EYG, JAK2 PDB-3FUP, JAK3 PDB-3LXK). For CK2, we selected the holoenzyme with ATP (PDB ID: 1JWH, 3.10 Å) [23]. The crystal structures were prepared for docking [remove water, add hydrogens, clean structure, and the default minimization (with ligand in place)] using Yasara, version 23.12.24 (YASARA Biosciences GmbH, Vienna, Austria) [24]. Ligands were either extracted from the PDB files or built in Spartan’24, version 24.1.1.0 (Wavefunction, Inc., Irvine, CA, USA) [25], with all ligands minimized in Spartan’24 using MMFF [26]. The pdbqt docking files were prepared using AutoDock Tools, version 1.5.7, and docking was performed using AutoDock Vina, version 1.1.2 (Molecular Graphics Laboratory, La Jolla, CA, USA) [27] using an exhaustiveness of 500. All docking runs were performed three times using a flexible ligand and rigid protein. Calculated binding free energies were used to compare to experimental data. The docking poses were viewed using PyMOL, version 2.5.2 (Schrödinger, Inc., New York, NY, USA) [28] and compared to crystal structures where available to confirm the correct binding pose. The ligands were chosen based on binding data obtained from Sun et al. [29] and the Selleckchem.com website (https://www.selleckchem.com/; accessed date 5 August 2024) [30].

### 2.2. Determination of the Impact of 4HPR Treatment on Janus Kinase 1 (JAK1) and Casein Kinase 2 (CK2) Functional Activity

The impact of 4HPR on JAK1 and CK2 function was evaluated using the Promega ADP-Glo Kinase assay (Madison, WI, USA, JAK1) and New England Biolabs (Ipwich, MA, USA, CK2) using fixed, high-level substrate and ATP (0.1 mg/mL IRS-1 (JAK1) and 200 µM CK2 substrate), 50 µM ATP (both assays) with 10 ng/mL protein (JAK1) and 50 units (CK2) with varying levels of 4HPR.

### 2.3. Human Cell Lines

The premalignant lung epithelial cell line, HBEC-KTRL53 [31], which was graciously provided by Dr. John Minna, UT Southwestern, was used for the RNA-seq and RT-qPCR analyses. A 100% tumorigenic, LUSC-origin (ATCC NCI-H520) cell line was employed for the RT-qPCR and the in vivo tumor studies. Cell lines were authenticated (STR profiling, Johns Hopkins Genetic Resources) and cultured in their optimal medium [keratinocyte serum-free medium (Invitrogen, Carlsbad, CA, USA) and RPMI-1640 medium (Thermo Fisher, Waltham, MA, USA) with growth factors (HBECKTRL53) and 5% heat-inactivated fetal bovine serum (H520), respectively].

### 2.4. Evaluation of 4HPR and TCZ Treatment on the Transcriptome of Premalignant Human Lung Epithelial HBEC-KTRL53 Cells

HBEC-KTRL53 cells were treated with low and high doses of 4HPR (0.5 and 1.0 µM) and TCZ (1 µg/mL and 2.5 µg/mL) singularly and in combination for 24h. Following treatment, total RNA was extracted (RNAprotect cell reagent and RNeasyPlus, Qiagen, Germantown, MD, USA). Data were analyzed using Qiagen Ingenuity Pathway Analysis software 2024. RT-qPCR analyses were conducted for select genes of high importance to the canonical pathways, which were determined to be significantly altered by 4HPR-TCZ treatment, e.g., immunogenic cell death, RHO GTPases (see Supplemental Table S1).

RNA was isolated using a PureLink RNA kit (Thermo Fisher Scientific, Waltham, MA, USA), converted to cDNA (Superscript IV FirstStrand Synthesis System, Thermo Fisher Scientific), and RT-qPCR was conducted (Power SYBR Green PCR Master Mix, 40 cycles, QuantStudio3 Real-Time PCR Systems, Thermo Fisher Scientific) using primer pairs designed by Invitrogen Primer Designer. The RT-qPCR primer sequences are contained in Supplemental Table S2. Target gene expression levels were calculated as Cts after subtracting housekeeping gene GAPDH Ct values.

### 2.5. Impact of TCZ on IL6-Mediated STAT3 Activation and Nuclear Translocation

HBEC-KTRL53 and H520 cells were cultured in growth factor-free base medium for 24 h. One hour prior to IL6 challenge (100 ng/mL), some of the cells were treated with the IL6 R inhibitor, tocilizumab (TCZ, 2.5 µg/mL). Thirty minutes after the addition of IL6, cells were harvested for immunoblotting (pSTAT3, Cell Signaling, Danvers, MA, USA) (cytosolic protein) or nuclear STAT3 (nuclear protein).

IL6 production was evaluated via an IL6 ELISA (R&D Systems, Minneapolis, MN, USA) with results expressed as pg/10^6^ cells. The impact of 4HPR (5 µM, complete medium) and singular (TCZ, 10 µg/mL) and combined 4HPR-TCZ (5 µM, 10 µg/mL, respectively, base medium) on PD-L1 protein expression (immunoblotting) was assessed. RT-qPCR analyses were conducted to further assess changes noted in IL6 pathways during the RNA-seq analyses.

### 2.6. Formulation and Characterization of the 4HPR-TCZ Human Serum Albumin-Chitosan Janus Nanoparticles

Janus particles (JNP-CTR) comprising two distinct compartments, human serum albumin (HSA) (Sigma Aldrich, St. Louis, MO, USA) and glycol chitosan (GC) (MedChemExpress, Monmouth Junction, NJ, USA), were fabricated using electrohydrodynamic (EHD) co-jetting, following protocols previously reported, with slight modifications. Briefly, jetting solutions of (1) 0.3% *w*/*v* solution of HSA and O, O′-bis [2-(N-succinimidyl-succinylamino)ethyl]polyethylene glycol (Sigma Aldrich, St. Louis, MO, USA) at 10% *w*/*w* in 20% ethanol-water and (2) 0.7% *w*/*v* solution of GC, 0.3% *w*/*v* of HSA, and 10% *w*/*w* of poly(ethylene glycol) diglycidyl ether (Sigma Aldrich, St. Louis, MO, USA) in 20% ethanol-water were simultaneously pumped through two parallel 25G needles at a flow rate of 0.04 mL/h to ensure laminar flow and stable solution interfaces. Upon droplet formation at the needle tip, 6 kV was applied to generate an electric field to induce the formation of a Taylor cone, subsequently developing an electrohydrodynamic jet. The jet broke into finely charged droplets, which underwent rapid solvent evaporation and solidification as they traveled toward the collector’s surface. The resulting solid particles were placed in an incubator at 37 °C for at least one week to ensure full crosslinking. Crosslinked particles were collected using a 0.01% (*v*/*v*) Tween 20 (Sigma Aldrich, St. Louis, MO, USA)-water solution, and serial centrifugation was carried out to obtain particles at the targeted size. Fluorescently labeled JNP-CTR for bicompartmental characterization was fabricated by replacing 5% *w*/*w* of the HSA in solution with albumin from bovine serum and Alexa Fluor 488 conjugate (Thermo Fisher Scientific, Waltham, MA, USA) in one compartment, and albumin from bovine serum and Alexa Fluor 647 conjugate (Thermo Fisher Scientific, Waltham, MA, USA) in the other compartment.

Similarly, particles that were partially loaded with only 4HPR (JNP-4HPR) and dually loaded Janus nanoparticles (JNP-4HPR/TCZ) were prepared with slight modifications to the original formulation. For JNP-4HPR, the HSA in the protein solution was substituted with a previously reported high-pressure homogenized fenretinide (HPH-4HPR) (0.3% *w*/*v*) [32]. For JNP-4HPR/TCZ, the HSA of the protein compartment was substituted with HPH-4HPR, and the HSA in the GC compartment was replaced by tocilizumab (TCZ) (Genentech, San Francisco, CA, USA).

Scanning electron microscopy (SEM) was used to characterize the geometric properties of the jetted Janus nanoparticles. The micrographs were obtained by FEI Nova 200 Nanolab SEM/FIB at the Michigan Center of Materials Engineering with an accelerating voltage of 5 kV and a current of 0.40 nA. ImageJ 1.54i. 2024 (Wayne Rasband, NIH, >5000 JNP/sample) was used to process and analyze the images to obtain key geometric properties: circularity, anisotropy, roundness, size, and size distribution [33].

Structured illumination microscopy (SIM) by Zeiss LSM 980 Airyscan2 Confocal + Superresolution Microscope (Zeiss, Oberkochen, Germany) was used to confirm the bicompartmental nature of fluorescence-labeled JNPs. JNPs were collected and drop-cast onto a glass slide for imaging. The confocal images were processed using ImageJ (1.54i. 2024) for improved visualization and to facilitate qualitative analysis [8].

After collection, JNPs were analyzed for particle size, polydispersity, and zeta potential using a Zetasizer Nano ZS (Malvern Panalytical Inc., Westborough, MA, USA) via DLS/ELS measurements. Reported values represent the average of triplicate measurements. Nanoparticle concentration was determined with a ZetaView^®^ TWIN NTA (Particle Metrix, Munich, Germany) through nanoparticle tracking analysis (NTA).

### 2.7. Encapsulation and Assessment of Bioactive 4HPR and TCZ Release from JNPs

The jetting yield of JNPs and the loading of TCZ were assessed using a Pierce 660 nm protein assay. Additional standards to account for TCZ and glycol chitosan were measured simultaneously, and a weighted-linear addition of the standard curve was used for the calculation.

The release profiles of TCZ and 4HPR were achieved through the centrifugation method. Briefly, the drug-encapsulated JNPs were suspended in phosphate buffer (pH = 7) and placed in a rotator at 37 °C. To access 4HPR release, a 5% ethanol phosphate buffer solution was added to ensure maximum solubility of the apolar molecule. At designated time points (1, 3, 6, 12, 24, 48, and 72 h), particles were spun down at an RCF of 21,300, and the resulting supernatant was removed. Fresh release buffer was added to the pelleted particles for further resuspension and incubation until the next time point. The extracted supernatants were analyzed via a tocilizumab (mAb-based) ELISA (IBL, Minneapolis, MN, USA). The total immunoreactive TCZ levels obtained from the ELISA reflect the cumulative release over time. For 4HPR, UV-vis spectroscopy was used to determine the cumulative release.

### 2.8. Confirmation of JNP-Released TCZ Bioactivity

A human IL-6Rα DuoSet ELISA (R&D Systems, Minneapolis, MN, USA) was used to measure TCZ bioactivity after release from 4HPR-TCZ JNP. A pharmaceutical stock solution TCZ (ACTEMRA^®^) served as the positive control. JNP-released TCZ samples at concentrations of 0.05 µg/mL and 0.25 µg/mL, along the control samples, and blank samples, were mixed with the IL-6R⍺ at 37 °C for an hour before well-plate loading. Bioactivity was assessed by measuring the percentage reduction of IL-6R⍺ binding levels relative to the control (no treatment). Previous investigations confirmed the capacity of JNP-released 4HPR to activate the execution phase caspase 3 enzyme activity [32].

### 2.9. Impact of 4HPR and TCZ Following IL6 Challenge on PD-L1 Intracellular Localization

First, the endogenous production of IL6 was evaluated in sera-starved H520 and HBEC-KTRL53 cell lines using an IL6 ELISA (R&D Systems, Minneapolis, MN, USA), with results reported as pg/10^6^ cells. Log-growth NCI-H520 and HBEC-KTRL53 cells were transferred to serum/growth factor-free media for 24 h. Next, the cells received a 30 min pretreatment (when indicated) with 4HPR and TCZ, followed by a IL6 challenge for 24 h. The experimental groups included the following: (1) control, mock treatment, (2) 5 µM 4HPR, (3) 20 µg/mL TCZ, (4) 5 µM 4HPR + 20 µg/mL TCZ, (5) 200 ng/mL IL6, (6) 5 µM 4HPR + 200 ng/mL IL6, (7) 20 µg/mL TCZ + 200 ng/mL IL6, and (8) 5 µM 4HPR + 20 µg/mL TCZ + 200 ng/mL IL6. After 24 h, cell membranes and nuclei were isolated using a cell fractionation kit (#9038, Cell Signaling, Danvers, MA, USA), protein levels recorded, followed by immunoblotting (PD-L1 Mab, Abcam, Waltham, MA, USA). As the levels of nuclear PD-L1 in the H520 cells were insufficient for detection by immunoblotting, a PD-L1 ELISA (DY156, R&D Systems, Minneapolis, MN, USA) was conducted with results reported as pg/10^6^ cells.

### 2.10. In Vivo Chemoprevention Studies

NCI-H520 cells [1 × 10^7^ suspended in 100 µL Matrigel™ (Corning Matrigel Matrix, San Diego, CA, USA)] were injected into the flanks of female BALB/c nude mice. Male BALB/c mice (*n* = 10, Ohio State Institutional Animal Care and Use Committee-approved) were obtained from Charles River (Germantown, MD, USA). Twenty-four hours post-cell implantation, intratumor treatments (all in 50 µL PBS) were as follows: (1) control (drug-free JNP), (2) JNP-4HPR (5 µM 4HPR release/96 h), and (3) JNP-4HPR/TCZ (5 µM 4HPR+1.2 µg TCZ release/96 h). Intratumor injections were given every 96 h for a total of 6 treatments. Mice were euthanized 24 h following the last treatment. Tumor tissues were then excised, and a caliper was used to determine the longest dimension (length) and largest perpendicular dimension (width), They were then fixed with 4% paraformaldehyde overnight, processed, paraffin-embedded, and sectioned for light microscopic and immunohistochemical analyses. Tumor volume (V) was then estimated by V = 0.5 × L × W2 [34]. The tumor markers evaluated by immunohistochemistry were as follows: Ki67 (tumor cell proliferation, Abcam, Walthan, MA, USA), cleaved caspase-3 (confirmation of apoptotic cells, Cell Signaling, Danvers, MA, USA), ERG (endothelial cell delineation, Abcam, Walthan, MA, USA), and PD-L1 (Abcam, Cambridge, MA, USA) with concurrent conduction of appropriate positive and negative controls. The impact of 4HPR and TCZ released by the JNPs on tumor growth indices and PD-L1 expression were determined by conduction of a complete scan and image capture of the H&E and IHC stained slides [Leica Dmi8 microscope with Leica ICC50W camera (Leica Application Suite X, Buffalo Grove, IL, USA)], followed by quantitative image analysis of the LUSC tumor tissue [ImagePro software (Media Cybernetics, Rockville, MD, USA)]. Determination of the extent of intra-tumor necrosis was conducted on image-captured complete tumor scans (Leica Dmi8 microscope) for identification of the region of interest (vital tumor area), followed by use of the Smart Segmentation tool to delineate the necrotic and viable tumor based on color, intensity, and background quantitative image analysis (ImagePro). These data are expressed as total tumor surface area and the percentage of tumor area that is necrotic.

*Statistical Analyses.* All data were initially evaluated for normality (Shapiro-Wilk test) to determine whether parametric (optimal) or non-parametric statistical tests would be employed. The Qiagen Ingenuity Pathway analyses employed Fisher’s Exact Test to determine the *p*-value of overlap. The Mann-Whitney U test was employed to evaluate RT-qPCR gene expression changes. ANOVA followed by a Tukey’s post hoc comparison was used to analyze the IL6R ELISA, Ki-67 indices, the intratumor hemorrhage, the PD-L1 IHC data, and the tumor necrosis data. Tumor tissue IHC analyses for cleaved caspase-3 were evaluated using the Kruskal-Wallis ANOVA followed by Dunn’s post hoc test. For all statistical analyses, a *p* value of <0.05 was considered statistically significant. 

## 3. Results

### 3.1. 4HPR Functions as a Competitive Inhibitor for ATP Binding at the JAK1 and CK2 Active Sites and Inhibits Function of Both Kinases in a Dose-Dependent Fashion

**Figure 1:**
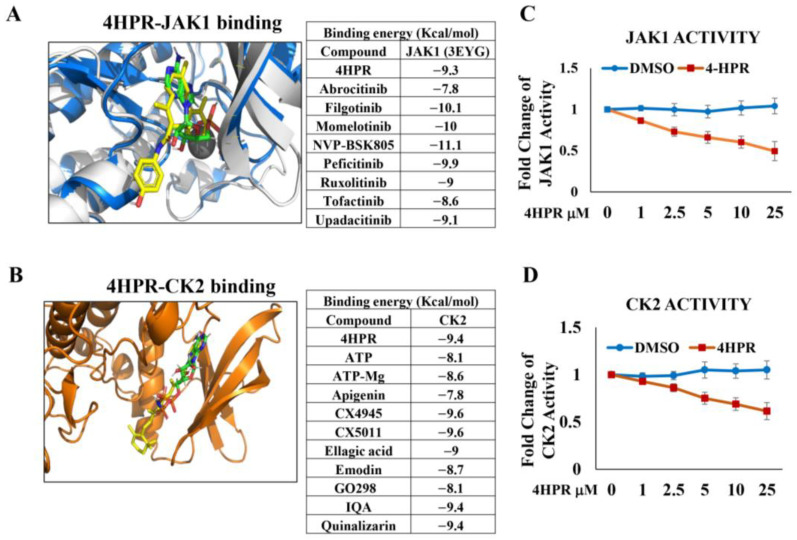
4HPR serves as a competitive inhibitor for ATP at the AK1 and CK2 active kinase sites. Molecular modeling was conducted to evaluate the capacity of 4HPR to function as a competitive inhibitor at the ATP-binding sites of two kinases, JAK1 (**A**) and CK2 (**B**), which are capable of phosphorylating and stabilizing PD-L1. Crystal structures were downloaded from the Protein Data Bank (http://www.rcsb.org/ accessed date: 10 July 2024) and compared using MOE. The crystal structures were prepared for docking (removing water, adding hydrogens, cleaning the structure, and performing default minimization with ligands in place) using Yasara. Ligands were either extracted from the PDB files or built in Spartan’24, with all ligands minimized in Spartan’24 using MMFF. Our data confirm that 4HPR functions as a competitive inhibitor, at levels comparable to enzyme-specific inhibitors, at the ATP binding site for both JAK1 and CK2 (4HPR-yellow, ATP-green in both images). Functional activity assays (**C**,**D**) confirm inhibition of kinase function at levels readily attainable by local delivery.

Molecular modeling studies (AutoDockVina, v1.1.2), crystal structures downloaded from Protein Data Bank http://www.rcsb.org (accessed 10 July 2024) confirmed that 4HPR binds at the JAK1 ATP binding site with a comparable binding affinity to established JAK inhibitors, e.g., upadacitinib and tofacitinib (Figure 1A). Molecular modeling of CK2, a constitutively active enzyme associated with numerous tumor-enabling pathways, including stabilization of PD-L1 to facilitate immune evasion (Figure 1B), demonstrated that 4HPR binds at the enzyme active site with a 10-fold higher affinity (as determined by K_d_), relative to the endogenous ligand and essential cofactor, ATP. Additional molecular modeling studies confirmed that 4HPR showed comparable binding energy for all 3 JAK isoforms relative to JAK-specific inhibitors (See Supplemental Figure S1).

Kinase functional activity assays (*n* = 3, ±S.D. for both JAK1 and CK2) show 4HPR (30 min pretreatment) demonstrated a dose-dependent inhibitory effect on JAK1 (Figure 1C) and CK2 (Figure 1D) kinase function. 4HPR levels that are readily achievable by local delivery (≈5 µM) show approximately 40% JAK1 and CK2 inhibition.

### 3.2. Combined Treatment with 4HPR and TCZ Reduced Expression of Pro-Proliferative Genes with Concurrent Increased Expression of Immune-Enhancing Genes in Premalignant Lung (PML) (HBEC-KTRL53) Cells

**Figure 2:**
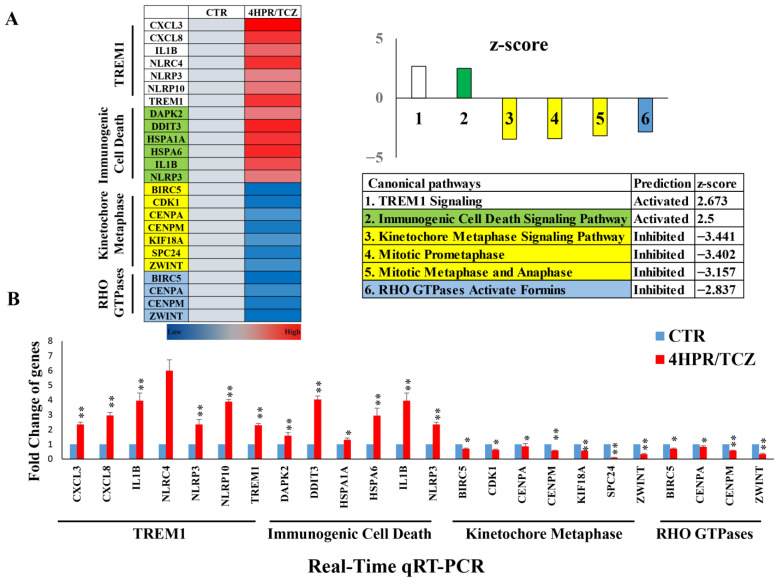
Combined 4HPR-TCZ treatment modulated premalignant lung epithelial cell gene expression towards reduced proliferation and increased immunogenic cell death. HBEC-KTRL53 cells were treated with 4HPR-TCZ (1 µM, 2.5 µg/mL 4HPR and TCZ, respectively) for 24 h, followed by RNA extraction and evaluation using Qiagen Ingenuity Pathway Analysis software. These studies showed that 4HPR-TCZ uniformly suppressed canonical pathways associated with cell cycle progression, cell proliferation, and cytoskeletal interactions, with concurrent upregulation of genes associated with immunogenic cell death pathways (**A**). Concurrent RT-qPCR analyses were carried out to evaluate select genes of high importance to the canonical pathways, demonstrating expression changes that aligned with the RNA sequencing data (* *p* < 0.05, ** *p* < 0.010, Mann-Whitney U 2-tailed test) (**B**).

Although single-agent and lower dosing levels of 4HPR and TCZ impacted gene expression, these effects were relatively insignificant. Differential gene expression data (Qiagen Ingenuity Pathway Analysis software) revealed that 4HPR-TCZ (1 µM, 2.5 µg/mL 4HPR and TCZ, respectively) uniformly suppressed canonical pathways associated with cell cycle progression, and proliferation (Figure 2A) The 4HPR-TCZ inhibition of gene expression also extended to RHO GTPases. These small signaling proteins contribute to cell cycle progression, in addition to actin-microtubule cytoskeleton interactions, cell migration, and vesicle trafficking [35]. 4HPR-TCZ also upregulated the expression of immunogenic cell death pathways that modulate cell surface composition and release soluble mediators that activate dendritic cell antigen processing to T cells [36,37]. Although TREM1 activation can induce pro- and antitumorigenic effects, TREM1 immunostimulatory signaling includes innate immune response activation, dendritic cell maturation, antigen presentation, and recruitment of NK and CD8+ T cells [38,39]. Subsequent studies that included 4HPR-TCZ treatment (1 µM, 2.5 µg/mL 4HPR and TCZ, respectively) of HBEC-KTRL53 cells (Figure 2B), followed by RT-qPCR analyses, confirmed significant expression changes that align with the RNA seq data in genes associated with these canonical pathways. The expression changes of each gene relative to its nontreated control were as follows: (1) TREM1 pathway: CXCL3 (+2.3), CXCL8 (+3.0), IL1B (+4.0), NLRC4 (+6.0), NLRP3 (+2.4), NLRP10 (+3.9); and TREM1 (+2.3); (2) immunogenic cell death pathway: DAPK2 (+1.6), DDIT3 (+4.0), HSPA1A (+1.3), HSPA6 (+3.0), IL1B (+4.0), and NLRP3 (+2.4); (3) Kinetochore Mataphase pathway: BIRC5 (−0.87), CDK1 (−0.62), CENPA (−0.83), CENPM (−0.57), KIF18A (−0.55), SPC24 (−0.08), and ZWINT (−0.33); (4) RHO GTPases pathway: BIRC5 (−0.70), CENPA (−0.83), CENPM (−0.57), and ZWINT (−0.33). These similar RT-qPCR data helped to substantiate the RNA-seq findings: * *p* < 0.05, ** *p* < 0.010.

RNA-seq upstream regulator analyses (with upstream regulator defined by Qiagen as “any molecule that influences expression, transcription or phosphorylation of another molecule”), followed by qRT-PCR analyses of additional 4HPR-TCZ-treated HBEC-KTRL53 cells, revealed gene expression changes consistent with reduction in cell cycle progression, DNA replication, proliferation, chromosomal transport, anti-apoptotic proteins, and angiogenesis, along with upregulation in cell cycle checkpoint inhibitors, augmentation of immune responsiveness, and apoptosis (Supplemental Figure S2). The qRT-PCR data were organized into four upstream regulator categories: cell cycle; IL-1β-mediated inflammation and immune regulation; TNF-mediated inflammation, immune response, and cell fate; and CEBPB-related growth modulation and regulation of gene expression. Expression trends observed in the upstream regulator RNA-seq data were recapitulated in the qRT-PCR analyses.

### 3.3. Pretreatment with 4HPR and TCZ Reduced STAT3 Signaling, Nuclear Translocation, and PD-L1 Levels and Localization in Human PML and LUSC Cells

**Figure 3:**
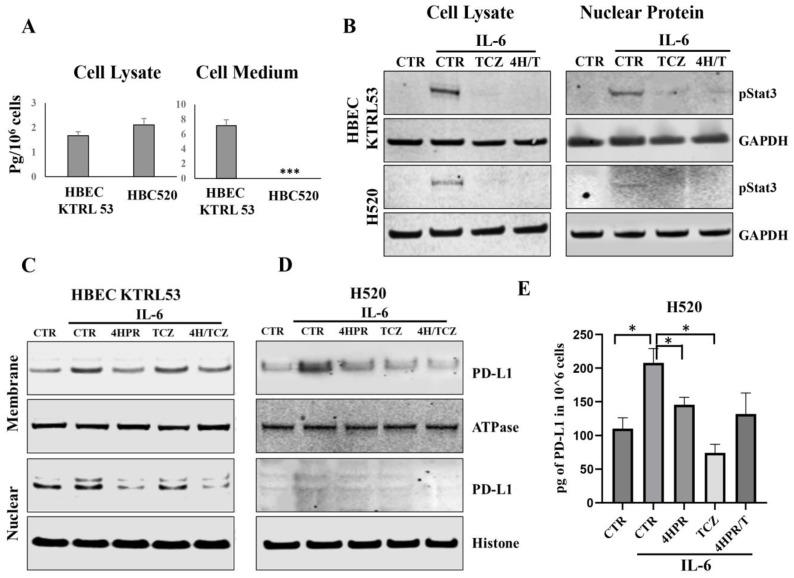
TCZ inhibits IL6-mediated STAT3 activation and nuclear translocation in target human PML and LUSC cells. (**A**). ELISA analyses on the H520 and HBEC-KTRL53 cell lines cultured in base medium demonstrated modest intracellular IL6 protein production and confirmed that only the premalignant cells released negligible IL6 to the extracellular milieu. *** *p* < 0.001. Log-growth HBEC-KTRL53 and H520 cells were cultured in base (growth factor-free medium), challenged with IL6 (100 ng/mL) for 30 min, followed by harvesting of cytosolic and nuclear proteins. Some cultures were pretreated with TCZ (10 µg/mL), 4HPR (5 µM), and TCZ-4HPR (10 µg/mL, 5 µM. respectively) for one hour prior to IL6 challenge. (**B**). The addition of IL6 resulted in phosphorylation of cytosolic STAT3, in addition to nuclear STAT3 translocation, while the addition of TCZ or 4HPR-TCZ abrogated STAT3 activation. (**C**,**D**). Cell fractionation studies to assess cellular PD-L1 localization following IL6 challenge with or without 4HPR, TCZ, or 4HPR+TCZ treatment showed that 4HPR and 4HPR-TCZ reduced mPD-L1 and nPD-L1 in both cell lines, while only the H520 malignant cells responded to solitary TCZ. (**E**). As nuclear PD-L1 were not clearly discernable by immunoblotting, PD-L1 ELISA was used to assess total PD-L1 levels. * *p* < 0.05.

While both cell lines produced IL6, the levels were modest and only increased slightly during growth in optimal medium. Also, only the PML cell line (HBEC KTRL53) released detectable IL6 levels (Figure 3A). Both the HBEC-KTRL53 and the H520 cells were responsive to IL6 challenge, as demonstrated by STAT3 phosphorylation and STAT3 nuclear translocation. Introduction of TCZ inhibited STAT3 activation, and addition of 4HPR did not antagonize this effect (Figure 3B). In contrast, the addition of cell line-specific growth factors [keratinocyte growth supplement (HBEC-KTRL53), FBS (H520)] failed to activate STAT3 signaling. Keratinocyte growth supplement (Invitrogen) contains two established STAT3 activators, i.e., recombinant human insulin-like growth factor and epidermal growth factor (EGF). While FBS is less chemically defined, it contains an abundance of interleukins, in addition to numerous STAT3-activating growth factors, including EGF, PDGF, and IGF-1, all of bovine origin. As IL6-mediated STAT3 signaling is known to activate PD-L1 expression, subsequent studies were conducted to assess the impact of TCZ and 4HPR pretreatment on both total PD-L1 and membrane (mPD-L1) and nuclear (nPD-L1) localization following IL6 challenge. Cell fractionation studies (Figure 3C,D) to assess cellular PD-L1 levels and localization following IL6 challenge with or without 4HPR, TCZ, or 4HPR+TCZ pretreatment showed that 4HPR and 4HPR-TCZ pretreatment reduced both mPD-L1 and nPD-L1 in the HBEC-KTRL53 cells, while 4HPR reduced overall PD-L1 in the H520 cells. Solitary TCZ pretreatment resulted in a higher overall PD-L1 reduction in the H520 cells. As H520 nuclear PD-L1 levels were not clearly discernable by immunoblotting, PD-L1 ELISA was used to assess pretreatment impact on total PD-L1 levels (Figure 3E).

### 3.4. JNP-4HPR/TCZ Characterization Confirmed a Low Polydispersity Index, Excellent Nanoparticle Stability in Suspension, Controlled and Sustained Agent Release, and Retention of Bioactivity

We then developed a novel JNP formulation for the co-delivery of 4HPR and TCZ. The JNPs evaluated in these studies were designed with an eye on future clinical application, i.e., aerosolized local delivery to the lung. Relative to synthetic polymers, biologic-based JNPs are biocompatible, biodegradable, nontoxic, nonimmunogenic, and can be prepared without the use of toxic chemicals or organic solvents, which are optimal aspects to reduce lung irritation. Accordingly, the JNP formulations comprised human serum albumin (HSA, 4HPR) and glycol chitosan (GC, TCZ). The GC-HSA Janus nanoparticles were chemically crosslinked and therefore stable, a critical feature for withstanding the shear forces exerted during nebulization. Chitosan contributes mucoadhesive and penetration-enhancing properties that would facilitate retention and uptake at the targeted mucus-covered upper-airway Type I epithelial cells.

**Figure 4:**
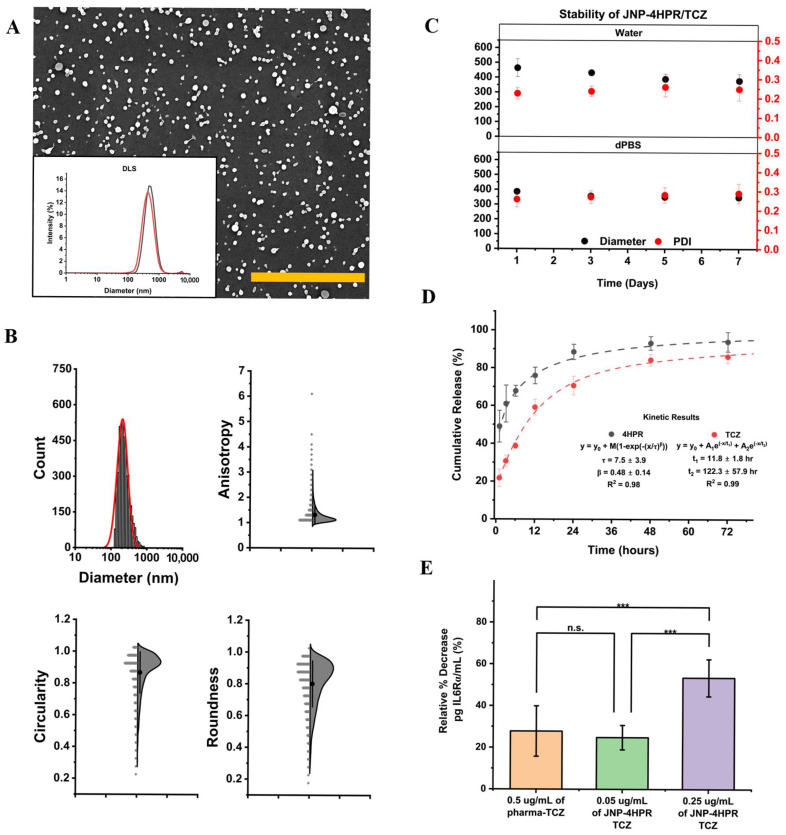
Engineered Janus nanoparticles provide a steady release of bioactive TCZ and 4HPR. Janus nanoparticle characterization study results. (**A**) Scanning electron micrographs of JNP-drug free control (JNP-CTR), JNP-4HPR, and JNP-4HPR-TCZ. Scale bar = 5 µm. The insets show the size distribution of the particles in suspension; red depicts JNPs suspended in dPBS, while black shows JNPs suspended in water. (**B**) JNP characterization study results that depict nanoparticle diameter, anisotropy, circularity, and roundness. (**C**) The data show consistency in diameter and minimal changes to the polydispersity index (PDI ≤ 0.3) of JNP-4HPR/TCZ over 1 week in water and dPBS. (**D**). The JNP kinetic release profile of 4HPR and TCZ, fitted to 2-phase exponential decay and cumulative Weibull, *n* = 3 within 4 days. (**E**) IL6R ELISA was employed to determine whether the TCZ released from 4HPR-TCZ JNP retained bioactivity. Our data show that JNP-released TCZ retained the ability to bind to the IL6R at a comparable level as pharmaceutical-grade TCZ. Furthermore, the JNP-released TCZ exhibited a dose-dependent effect and the concurrent release of 4HPR did not antagonize TCZ function. *** *p* < 0.001.

We used EHD co-jetting to prepare all JNPs. In brief, two distinct jetting solutions were prepared that contained either 0.3% *w*/*v* HSA, O,O′-bis [2-(N-succinimidyl-succinylamino)ethyl]polyethylene glycol (10% *w*/*w*), or 0.7% *w*/*v* GC, 0.3% *w/v* HSA, and poly(ethylene glycol) diglycidyl ether (10% *w*/*w*). An ethanol-water mixture was used for both cases. Upon droplet formation at the needle tip, an electrical potential of 6 kV was applied to induce the formation of a Taylor cone. JNPs were collected in a 0.01% (*v*/*v*) Tween 20-water mixture and subsequently fractionated using serial centrifugation. Geometric factor analysis of the scanning electron micrograph (Figure 4A,B, Supplemental Figures S3 and S4, Supplemental Table S3) of JNPs revealed high circularities (1 = perfect circle), low anisotropy (low = more spherical shape), and excellent roundness (1 = perfect sphere). JNP-4HPR/TCZ stability in suspension is crucial for drug delivery to moist environments, such as the lungs. DLS stability studies (Figure 4C) revealed consistency in diameter and minimal changes to the polydispersity index (PDI ≤ 0.3) of JNP-4HPR/TCZ over 1 week in water and dPBS. Confocal microscopy confirmed the bicompartmental nature of the JNP-4HPR/TCZ nanoparticles (Supplemental Table S3) to be true post-collection.

Next, we conducted release studies to establish the co-release of 4HPR and TCZ. The nanoparticle release models employed the best-fit application (Figure 4D). For the 4HPR loaded in JNPs, Fickian diffusion (where ß < 0.75) dominated the release of the apolar molecule from the protein matrix, where cumulative release of 91% was achieved within 4 days. For TCZ, our results show a cumulative release of 83% over 4 days, consistent with our previously reported value [7]. A fit to a two-phase exponential decay curve reveals a faster burst release of TCZ at the surface with a half-life of 3.6 h, and a slower release from within the particles with a half-life of 36.8 h. While the degradation of the particles can result in further release, our data (Figure 4C) show preservation of the particle integrity over seven days. As a result, we do not suspect that the particle degradation is the release mechanism for TCZ.

Moreover, results from the IL6R ELISA revealed that TCZ released from the JNP-4HPR/TCZ exhibited higher inhibition of IL6R binding compared to the pharmaceutical TCZ. In addition, JNP-released TCZ demonstrated a positive dose responsiveness, with greater inhibition noted at higher dosing levels (Figure 4E). Previous studies have confirmed the bioactivity of 4HPR-releasing nanoparticles. The JNP-4HPR/TCZ release profile demonstrates a steady release over time, which is optimal for in vivo local delivery efficacy as drug levels will be sustained.

### 3.5. Local Delivery of TCZ-HPR JNPs Inhibited NCI-H520 LUSC Tumorigenesis In Vivo

**Figure 5:**
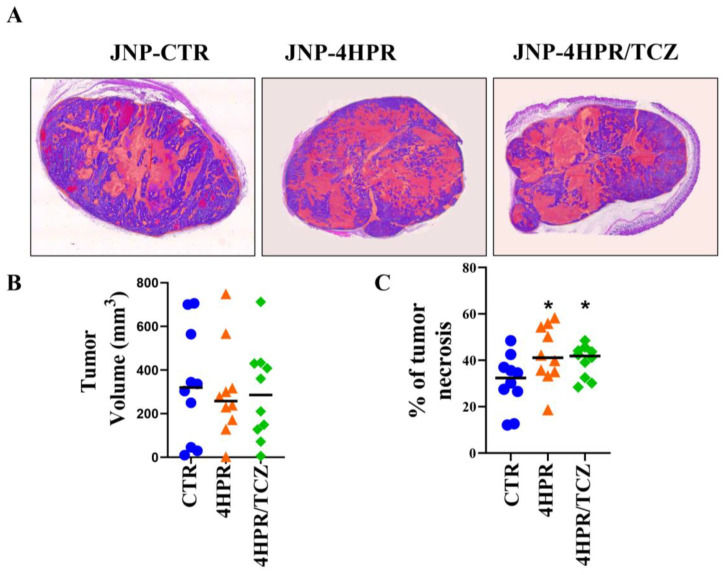
Local delivery of TCZ-HPR JNPs inhibited NCI-H520 LUSC tumorigenesis in vivo. While tumor external measurements (V = 0.5 × L × W^2^) were comparable among all experimental groups (control drug-free JNPs, fenretinide (4HPR)-releasing JNPs, 4HPR+tocilizumab (TCZ)-releasing JNPs) (**A**,**B**). Histopathologic assessment revealed that JNP-4HPR- and JNP-4HPR-TCZ-treated tumors showed significantly greater intratumor necrosis (**A**,**C**) (ANOVA, Tukey’s post hoc test, *n* = 10 every group, * *p* < 0.05) relative to the control, drug-free tumors. Consequently, the tumor volume was significantly decreased in the JNP-4HPR- and JNP-4HPR-TCZ-treated groups.

All mice that received H520-Matrigel injections formed tumors of comparable external sizes (Figure 5A,B). Treatment with JNP-4HPR and JNP-4HPR/TCZ, however, significantly increased intra-tumor necrosis, at * *p* < 0.05 [32% in the control group, 40% in the 4HPR-treated group, and 41% in the 4HPR-TCZ-treated group], thereby significantly reducing viable tumor volume in the treated relative to control tumors (see Figure 5A,C).

**Figure 6:**
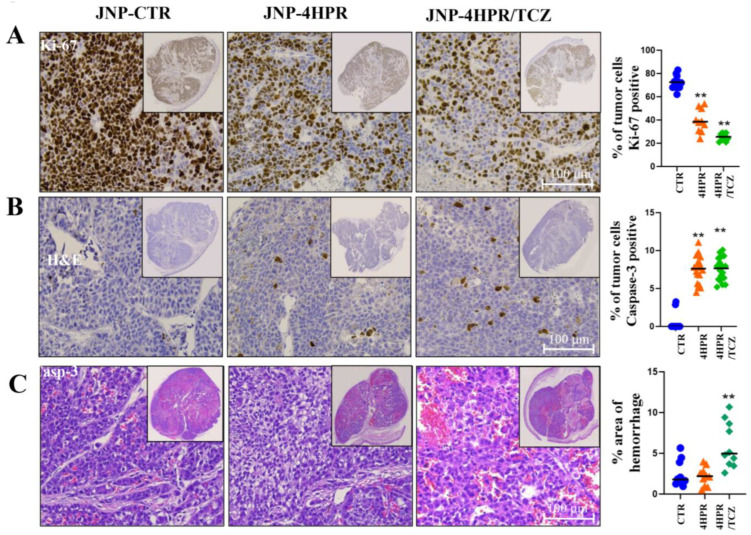
Local delivery of TCZ-HPR JNPs inhibited NCI-H520 LUSC tumorigenesis in vivo. Although all mice that received H520-Matrigel injections formed tumors, 4HPR JNP, and to a greater extent, 4HPR+TCZ JNP, significantly disrupted tumor cell growth and angiogenesis ((**A**–**C**), *n* = 10, ANOVA with Tukey’s post hoc test). Control tumor cells exhibited very high proliferation indices ((**A**), Ki67 labeling average 80%), with significantly reduced proliferation noted in the 4HPR and 4HPR-TCZ-releasing JNP-treated groups (** *p* < 0.01). Overall, the H520 were highly apoptosis-resistant (**B**). Tumors in the control, drug-free JNP mice demonstrated negligible apoptosis (≈1% overall tumor cells), as identified by nuclear staining for the executive phase, caspase-3. The introduction of 4HPR JNP and 4HPR-TCZ JNP significantly increased casp-3 (** *p* < 0.01) to approximately 30% in the 4HPR-JNP and 40% 4HPR+TCZ-JNPs, respectively. 4HPR-TCZ JNP (**C**) also significantly increased vascular instability (** *p* < 0.01), as observed by the extent of extravasated erythrocytes in dual-treated tumors.

In addition, the control tumor cells exhibited exceptionally high proliferation indices (average Ki67 labeling of 80%), were highly apoptosis-resistant [<1% cleaved caspase-3 (casp-3)], and exhibited highly infiltrative growth and vascular invasion (see Figure 6). In contrast, tumors that received JNP-4HPR and JNP-4HPR/TCZ exhibited significantly reduced proliferation indices, at ** *p* < 0.01 [Ki67 expression averages of 79% in the control group, 44% in the 4HPR-treated group, and 27% in the 4HPR-TCZ-treated group]. While intratumor apoptosis was not high in any tumor group, JNP-4HPR and JNP-4HPR/TCZ significantly increased intratumor nuclear staining of the execution phase caspase-3, at ** *p* < 0.01 [Caspase-3 expression averaged 0.6% in the control group, 7.1% in the 4HPR-treated group, and 7.5% in the 4HPR-TCZ-treated group]. The dually loaded JNPs also significantly increased vascular instability (** *p* < 0.01), as observed by the extent of extravasated erythrocytes in dual-treated tumors [2.1% in the control group, 2.5% in the 4HPR-treated group, and 5% in the 4HPR-TCZ-treated group]. Nuclear staining for pSTAT3 was negative in all tumor tissue.

Minimal parenchymal and stromal PD-L1 staining was observed in all three experimental groups (control, JNP-4HPR JNP, JNP-4HPR/TCZ). Small foci of intensely stained PD-L1 positive cells, primarily located at the periphery of the tumor islands and extending into the tumor stroma, were observed. While some of the PD-L1-expressing cells could represent murine macrophages or neutrophils, the monoclonal antibody employed (Abcam ab205921) is stipulated as specific for human samples (substantiated by Abcam-conducted advanced validation and knock-out validation assessments). These data imply that PD-L1 staining reflects immunoreactant human H520 cells. Tumor PD-L1 staining was primarily nuclear, with only occasional cells demonstrating cell membrane staining. Furthermore, many of the PD-L1-expressing tumor cells transitioned from an epithelioid to a spindled, EMT-like phenotype morphology.

## 4. Discussion

The focus for this study was two-fold. First, to determine if a combination of TCZ and 4HPR can collaboratively function toward the immunochemoprevention of LUSC and to elucidate the associated mechanisms. Secondly, to formulate and functionally evaluate in vivo Janus nanoparticles for combined local release of bioactive 4HPR and TCZ. Previous studies from our team have confirmed the benefits of controlled-release local delivery formulations compared to bolus drug delivery with regard to drug stability and bioavailability, which ultimately resulted in improved chemopreventive efficacy [6,21,32] Electrohydrodynamically co-jetted nanoparticles are very well suited for the preservation of complex protein tertiary structures, in addition to delivery of challenging, extremely hydrophobic agents [6,7,32].

IL6 is an integral factor in LUSC tumorigenesis. In addition to driving inappropriate proliferation and angiogenesis, IL6 sustains JAK/STAT3 signaling and impairs the local immune function via prevention of dendritic cell maturation, recruitment, and expansion of myeloid-derived suppressor cells and tumor-associated neutrophils and upregulation of immune checkpoint inhibitors, such as PD-L1 [40]. As an immediate IL6 downstream mediator, JAK1 functions in conjunction with the positive regulator of the JAK/STAT pathway, CK2, to initiate IL6 signaling [41]. Via phosphorylation of PD-L1 (JAK1 at Tyr112; CK2 at Thr290) and subsequent glycosylation, both JAK1 and CK2 promote PD-L1 stabilization and prevent degradation, which sustains PD-L1 levels and directly facilitates local immunosuppression [42]. Sustained PD-L1 membrane expression enables premalignant or malignant lung lesions to persist by masking neoantigens and avoidance of immunoprotective natural killer and cytotoxic T cells [43]. Our molecular modeling and kinase assay data show, for the first time, that 4HPR functions as a competitive inhibitor for ATP at the active sites of these key signaling and immune-modulating kinases, JAK1 and CK2. As would be anticipated by replacement of the essential cofactor, ATP, 4HPR binding impairs function of both kinases. Previous studies by our lab have confirmed the capacity of 4HPR to serve as a competitive inhibitor for active site ATP binding for several kinases upregulated during carcinogenesis, e.g., FAK, Pyk2, Wnt, and JNK [18,21]. This apparent 4HPR “drug pleiotropy” likely reflects the highly conserved nature of the ATP binding site in protein kinases [44] and the fact that 4HPR is a derivative of the naturally occurring micronutrient, vitamin A, which has been shown to inhibit cancer cell kinase activity [45]. With regard to immunochemoprevention, concurrent delivery of TCZ and 4HPR would harness the direct inhibition of the IL6 receptor by TCZ, combined with 4HPR inhibition of JAK1 and CK2 function. Collectively, these effects should appreciably reduce PD-L1 expression and stabilization in the local tumor milieu.

Deep learning-enabled studies on human tumor cells, which demonstrated the capacity of RNA-seq data to predict patient tumor cell responsiveness to the tested agents [46], motivated RNA-seq analyses on human premalignant lung (PML) epithelial cells. Collectively, the RNA-seq data confirm that combined 4HPR-TCZ treatment, dosed at local delivery achievable levels, significantly inhibited pathways associated with cell cycle progression and cell division, while concurrently upregulating immune response and exposure of damage-associated molecular patterns pathways [47]. Given the recognized 4HPR and TCZ mechanisms of action, these data are not surprising. Through its capacity to undergo redox cycling and induce oxidative stress, 4HPR is highly accepted as an inducer of apoptosis [48]. In addition, 4HPR has been shown to upregulate every pathway necessary to induce terminal differentiation in epithelial cells [20]. Both of these programmed cell death mechanisms halt cell cycle progression, remove cells from the proliferative pool, and eliminate the potential for cell transformation. Further, TCZ-mediated inhibition of IL6-STAT3 signaling would reduce the expression of genes essential for apoptosis resistance, proliferation, inflammation, and survival [49]. While the immunosuppressive impact of IL6 is thought to primarily manifest at the immune cell and microenvironmental levels, the observed upregulation of immune response pathways are consistent with a direct effect on the PML cells themselves.

While both the HBEC-KTRL53 and H520 cells produce IL6, only the HBEC-KTRL53 cells release detectable IL6. Relative to high IL6-producing cells (3000 pg/106 cells, human oral squamous cell carcinoma) [16], the premalignant and malignant lung cell lines used in our study only produced negligible levels of IL6. In contrast, a study that assessed IL6 levels in LUSC tumor tissue showed moderate to high IL6 levels in tumor cells and cancer-associated fibroblasts, findings that substantiate IL6 in vivo relevance [50]. As demonstrated by nuclear translocation of pSTAT3, both the premalignant HBEC-KTRLp53 cells and the LUSC H520 cells responded to IL6, findings that are consistent with retention of a functional IL6R and the gp130 signaling complex. The reduction in STAT3 activation and PD-L1 levels following 4HPR-TCZ pretreatment is consistent with disruption of the JAK-STAT3 signaling cascade, in addition to reduced PD-L1 post-translational stabilization.

For our in vivo, proof-of-concept studies, 4HPR and 4HPR-TCZ releasing JNPs were injected directly at the tumor implantation site. Previous tertiary chemopreventive in vivo studies from our team have confirmed that the controlled local drug release provided by JNPs is superior to local bolus drug delivery [6]. When JNPs are subjected to injection, protein adsorption and corona formation are potential concerns. Corona formation, however, was less of an issue in this study, as the particles were delivered intratumorally. In future applications, we envision aerosolizing these particles for pulmonary delivery. During aerosolization, mucin and pulmonary surfactant corona formation is anticipated, and we will address this issue once these studies are underway.

Consistent with the aggressive nature of this LUSC cell line, all mice injected with H520 cells formed tumors. While the measured tumor volumes were comparable among all experimental groups, tumor histology revealed statistically significant more intratumor necrosis and vital tumor tissue loss in the JNP-4HPR- and JNP-4HPR/TCZ-treated groups. JNP-released 4HPR and 4HPR-TCZ also significantly increased intratumor apoptosis (as detected by intranuclear caspase 3) and significantly decreased proliferation (nuclear Ki-67 labeling), most appreciably with dual 4HPR-TCZ treatment. Of note, the proliferation indices of the control tumors were exceptionally high (≈80% Ki-67 labeling), which indicates an increased demand for angiogenesis to prevent tumor hypoxia. We have previously shown that 4HPR significantly inhibits cancer cell matrix-type membrane metalloproteases, in addition to gelatinases (MMPs 2 and 9) [21], which are critical for cell invasion, stimulation of angiogenesis, and release of growth factors from the ECM [51]. These findings, therefore, likely reflect collective 4HPR-TCZ mechanisms of action, including induction of programmed cell death, reduction in IL6-STAT3 signaling, inhibition of angiogenesis, and interference with tumor and endothelial cell-ECM interactions. Our vascular instability data during 4HPR-TCZ JNP treatment suggest disruption of the endothelial cell-angiogenic cascade, which is a process highly dependent on regulated MMP activities [52].

In contrast to our findings, studies that employed the IL6 inhibitor, siltuximab, did not exhibit any cancer-inhibitory effects on LUSC tumor cell flank xenografts in nude mice [50]. These disparate results most likely reflect the agents employed and the method of drug delivery. Both drugs interfere with IL6, although via different mechanisms. TCZ suppresses IL6 signaling through binding to IL6R, while siltuximab binds to IL6 directly and is thought to convey a broader range of effects. Siltuximab was delivered three times a week via peritoneal injection. Although intraperitoneal delivery provides fast drug absorption, small-molecule drugs like siltuximab enter the portal vein and undergo first-pass metabolism, resulting in reduced systemic uptake [53]. Also, for these studies, TCZ was delivered in combination with a complementary mechanism of action agent, 4HPR. In addition, the locally delivered JNPs create a reservoir that provides a sustained release of bioactive and bioavailable drug directly to the target tissue.

Negligible membrane-associated PD-L1 expression was found in the tumor cells. While these findings could partially reflect TCZ-4HPR signaling inhibition, even control tumors lacked PD-L1 expression. The fact that these experiments used athymic T cell-deficient mice, which would result in the absence of a highly potent activator of the JAK/STAT PD-L1 upregulation pathway, i.e., interferon gamma, is a more plausible explanation [54]. When PD-L1 was identified, it was primarily intranuclear (nPD-L1), and seen in tumor cells following 4HPR treatment. Nuclear translocation of PD-L1 can have biphasic effects on tumorigenesis [55]. Tumor-promoting effects of nPD-L1 include activation of growth-promoting, pro-angiogenic pathways, DNA repair, and upregulation of immune checkpoint inhibitors. Conversely, nPD-L1 can also promote tumor suppression via increased MHC-I expression, which enhances antitumor immunity and induction of cellular senescence and programmed cell death via proptosis [55]. Additional studies are needed to further investigate agent impact on PD-L1 cellular localization.

## 5. Conclusions

These studies provide mechanistic insights regarding how combined nanoparticle-based delivery of 4HPR and TCZ can upregulate immunochemopreventive pathways via modulation of gene expression and suppression of tumor-promoting processes, such as angiogenesis and sustained proliferation. An immunoprevention shortcoming of these studies, however, was the use of T-cell-deficient athymic mice. Consequently, the in vivo studies were only able to assess the agents’ chemopreventive capacities. Proposed future studies, which will employ humanized immune system mice, will enable a more comprehensive assessment of the impact of 4HPR-TCZ Janus nanoparticles on the local tumor microenvironment, host immune cells, and tumorigenesis.

## 6. Patents

Susan R Mallery and Joerg Lahann are co-inventors of a patent entitled “Engineered Janus Nanoparticles for Field Coverage Chemoprevention of Lung Cancer”. The PCT was filed 2-28-25.

## Data Availability

The data that support the findings of this study are available from the corresponding author upon request.

## Supplementary Materials

The following supporting information can be downloaded at: https://www.mdpi.com/article/10.3390/pharmaceutics17111471/s1, Figure S1: 4HPR serves as a competitive inhibitor for ATP at the active kinase sites for all 3 JAK isoforms. Figure S2: RNA seq upstream regulator analyses reveal 4HPR-TCZ treatment significantly modulates upstream regulators. Figure S3: Bicompartmental Janus nanoparticles were ultilized this study. Figure S4: 4HPR increased nuclear PD-L1 in LUSC tumors in vivo. Table S1: Reported protein functions for genes assayed by rt-PCR. Table S2: RNA primer sets used for the rt-PCR studies. Table S3: Properties of the jetted and the suspended JNPs.
