# Supplementary material for: Engineered Fenretinide- and Tocilizumab-Releasing Janus Nanoparticles for Site-Directed Immunochemoprevention of Squamous Cell Carcinoma of the Lung
Source: Pharmaceutics. 2025 Nov 14;17(11):1471. doi: 10.3390/pharmaceutics17111471 (PMC12655460; doi:10.3390/pharmaceutics17111471)
Supplement: Supplementary file 1 [file pharmaceutics-17-01471-s001.zip › pharmaceutics-3939227-supplementary.pdf]

Engineered Fenretinide- and Tocilizumab-Releasing Janus Nanoparticles for Site-Directed Immunochemoprevention of Squamous Cell Carcinoma of the Lung

Daren Wang [1], Albert Chang [4,5], Fortune Shea [1], Yifei He [4], Richard Spinney [3], Jonathan D. Whitsett [1], Joerg Lahann [4,5,6,7] and Susan R. Mallery [1,2,*]

Supplemental Fig. 1

4HPR-JAK isoform binding

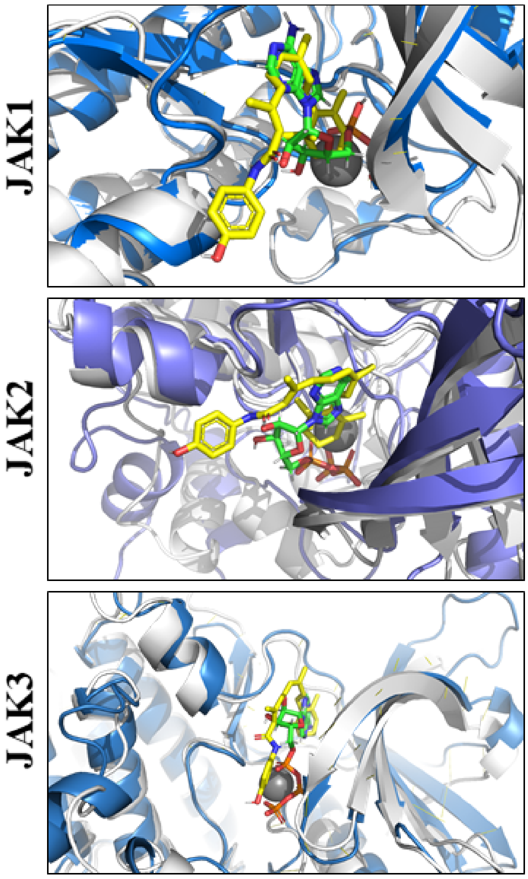

Compound	Binding E (Kcal/mol) JAK1 (3EYG)	JAK2 (3FUP)	JAK3 (3LXK)
4HPR	-9.3	-7.9	-8.5
Abrocitinib	-7.8	-8.1	-8.3
Filgotinib	-10.1	-9.4	-9.8
Momelotinib	-10	-9.3	-10.2
NVP-BSK805	-11.1	-10.9	-10.9
Peficitinib	-9.9	-8.6	-9.3
Ruxolitinib	-9	-8.8	-8.7
Tofactinib	-8.6	-8.1	-9
Upadacitinib	-9.1	-9.3	-9

Supplemental Figure 1. ***4HPR serves as a competitive inhibitor for ATP at the active kinase sites for all 3 JAK isoforms.*** Molecular modeling was conducted to evaluate the capacity of 4HPR to function as a competitive inhibitor at the ATP-binding sites of JAK1 (A), JAK2 (B) and JAK3 (C). Crystal structures were downloaded from the Protein Data Bank and were compared using MOE. The crystal structures were prepared for docking (remove water, add hydrogens, clean structure, and the default minimization (with ligand in place) using Yasara. Ligands were either extracted from the PDB files or built in Spartan2448, with all ligands minimized in Spartan'24 using MMF37. Our data confirm that 4HPR functions as a competitive inhibitor, at levels comparable to enzyme-specific inhibitors, at the ATP binding site for all 3 JAK isoforms. (4HPR-yellow, ATP-green in all images).

Supplemental Fig. 2

C

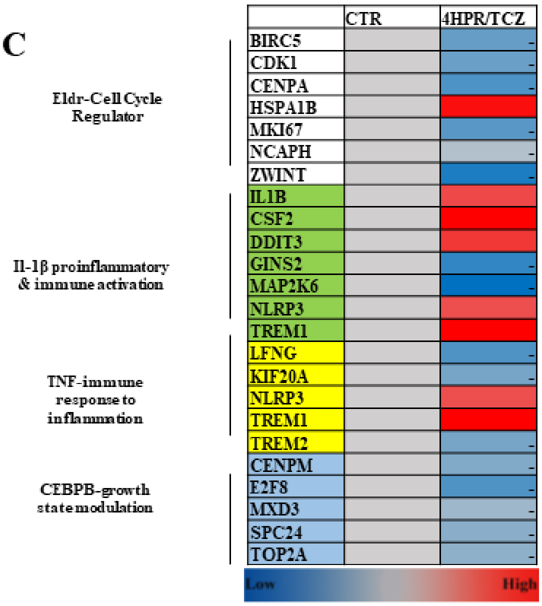

Upstream Regulators

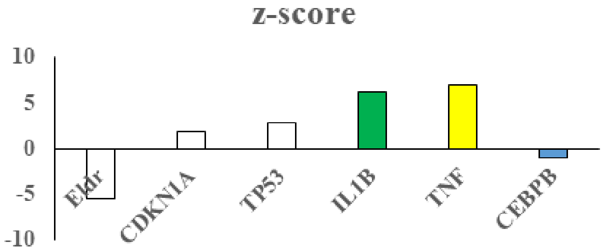

Upstream regulator	z-score
Eldr	-5.467
CDKN1A	1.908
TP53	2.816
IL1B	6.201
TNF	6.93
CEBPB	-1.085

D **Real-Time qRT-PCR**

Eldr-CDKN1A-p53 Cell Cycle	Il-1β inflammation & immune regulation	TNF-inflammation immune response & cell fate	CEBPB-growth state modulation & regulation of gene expression
BIRC5↓**	IL-1 B ↑*	LFNG↓**	CENPM↓ **
CDK1↓*	CSF2↑ *	KIF20A↓**	E2F8↓ **
CENPA↓*	DDIT3↑ **	NLRP3↑**	MXD3↓ **
HSPA1A↓**	GINS2↓**	TREM1↑**	SPC24↓ **
MKI67↓*	MAP2K6↓**	TREM2↓**	TOP2A↓ **
NCAPH↓**	NLRP3↑**		
ZWINT↓**	TREM1↑**		

Supplemental Figure 2. ***RNA seq upstream regulator analyses reveal 4HPR-TCZ treatment significantly modulates upstream regulators.*** HBEC-KTRL53 cells were treated with that 4HPR-TCZ (1µM, 2.5 µg/ml 4HPR & TCZ, respectively) for 24h, followed by RNA extraction and evaluation using Qiagen Ingenuity Pathway Analysis software. The Upstream Regulators and associated genes encompassed four functional categories i.e. Cell cycle, IL-1 cytokine, TNF/TNFR pathway, and Transcription factors. Accompanying qRT-PCR studies showed down regulation of genes associated with cell cycle progression, DNA replication, proliferation, chromosomal transport, anti-apoptotic proteins and angiogenesis with upregulated expression of cell cycle checkpoint inhibitors, augmentation of immune responsiveness, and apoptosis associated genes.

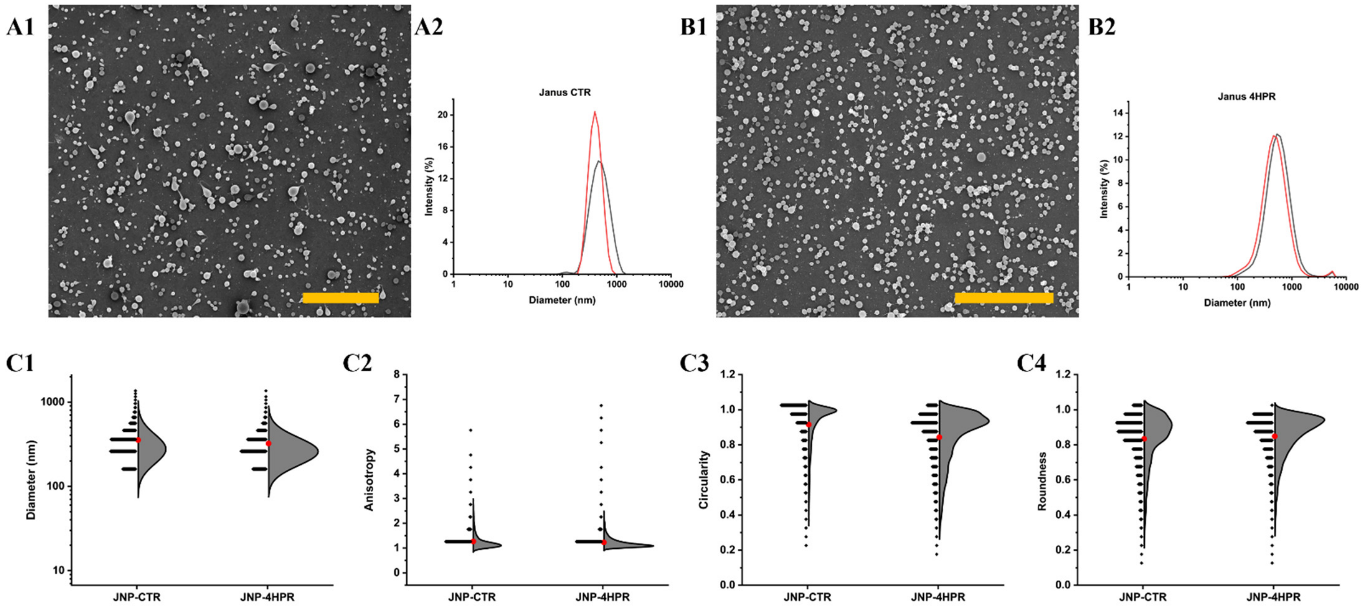

Supplemental Figure 3. ***Bicompartmental Janus nanoparticles were ultilized this study.*** (a1) is the 3D rendering of the fluorescently-tagged JNPs obtained from confocal microscopy (a2) zoom-in of a single JNP. Qualitative analysis of the fluorescence intensity profile where (a3) is on the xy plane and (a4) along the yellow projection in c2 of the z-axis; the green channel is glycol chitosan, and the magenta channel is the albumin compartment.

Supplemental Fig. 4

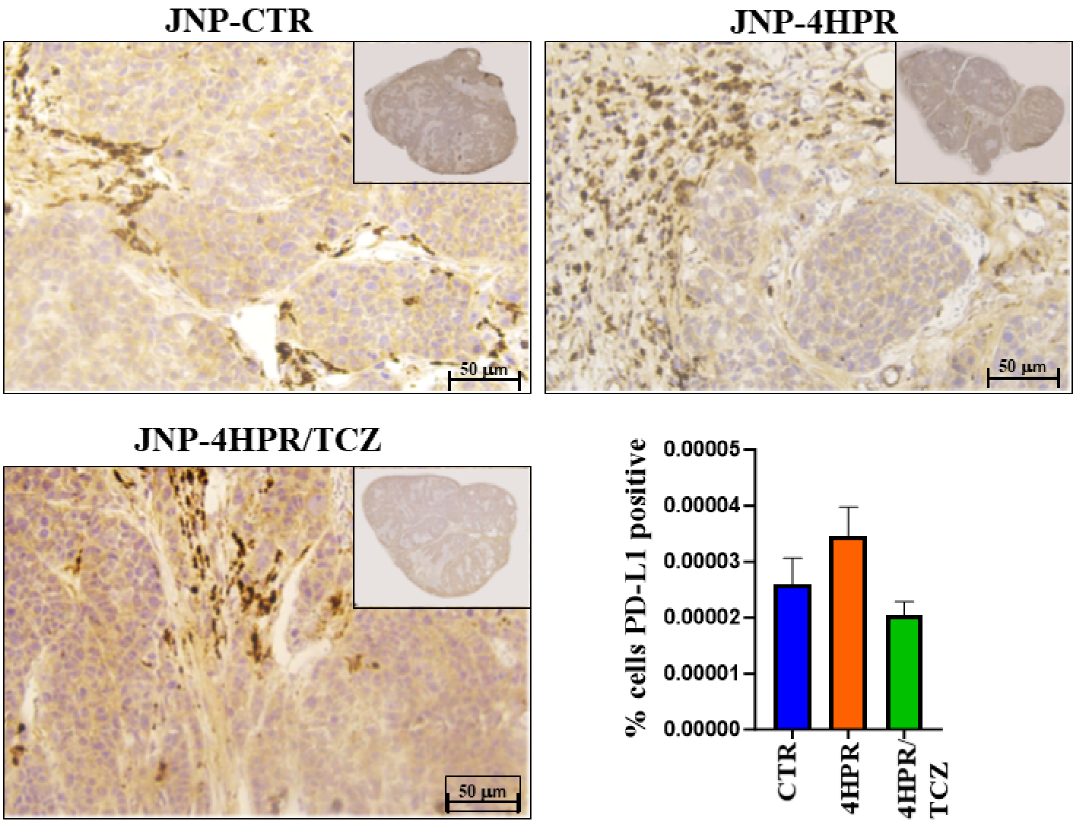

Supplemental Figure 4. ***4HPR increased nuclear PD-L1 in LUSC tumors in vivo.*** Overall, PD-L1 staining in all three groups (control, 4HPR, 4HPR-TCZ) was minimal. This finding likely represents the absence of a key initiator of PD-L1 expression i.e. interferon γ, as the murine hosts were T cell deficient. Small foci of intensely stained PD-L1 positive LUSC cells, primarily located at the periphery of the tumor nests and extending into the stroma, were identified. Many of these cells assumed a more spindled phenotype, consistent with a transition to an epithelial-myoepithelial phenotype. While focal membrane staining was noted, staining deposition was primarily intranuclear (nPD-L1). Although 4HPR administration increased nPD-L1 levels, no significant differences were noted among the tumor groups. A variety of cell stressors, including reactive oxygen species and the highly effective initiator, hypoxia, can induce PD-L1 expression. Provided the very high proliferation indices and the large areas of tumor necrosis in all groups, tumor hypoxia due to insufficient angiogenesis likely induced nPD-L1. The modest nPD-L1 increase observed with 4HPR may reflect 4HPR-redox active status.

Supplemental Table 1. Reported protein functions for genes assayed by rt-PCR.

**Protein**	**Functions**
ALDH3A1	Aldehyde Dehydrogenase 3 Family Member A1. Aldehyde dehydrogenases oxidize various aldehydes to the corresponding acids. They are involved in the detoxification of alcohol-derived acetaldehyde and in the metabolism of corticosteroids, biogenic amines, neurotransmitters, and lipid peroxidation.

AURKB	AURKB activation is associated with acquired resistance to EGFR TKIs, and that AURKB constitutes a potential target in NSCLC progressing to anti-EGFR therapy and not carrying resistance mutations https://www.nature.com/articles/s41467-019-09734-5
BIRC5	BIRC5 is an immune-related gene that inhibits apoptosis and promotes cell proliferation https://www.nature.com/articles/s41598-020-79736-7DNA
Bub1	Budding Uninhibited By Benzimidazoles 1 Homolog. The encoded protein functions in part by phosphorylating members of the mitotic checkpoint complex and activating the spindle checkpoint. This protein also plays a role in inhibiting the activation of the anaphase promoting complex/cyclosome.
CDK1	The protein encoded by this gene is an inner protein of the kinetochore, the multi-protein complex that binds spindle microtubules to regulate chromosome segregation during cell division.
CENPA	Centromere Protein A. This gene encodes a centromere protein which contains a histone H3 related histone fold domain that is required for targeting to the centromere. Diseases associated with CENPA include Crest Syndrome and Luminal Breast Carcinoma A.
CENPM	This gene encodes a cell surface tyrosine kinase receptor for members of the platelet-derived growth factor family. It plays a role in organ development, wound healing, and tumor progression.
CSF2	GM-CSF is a cytokine that controls the production, differentiation, and function of granulocytes and macrophages.
CXCL3	A secreted growth factor that signals through the G-protein coupled receptor. This protein plays a role in inflammation and as a chemoattractant for neutrophils.
CXCL8	A member of the CXC chemokine family and is a major mediator of the inflammatory response. The encoded protein is commonly referred to as interleukin-8 (IL-8)
CXCL10	Functions to increase accumulation of CVCR3+ effector T cells, particularly CD8+ T cells and facilitates antitumor activities either directly or via potentiation of effector CD4+ T cells.
DAPK2	Death Associated Protein Kinase 2. This protein contains a N-terminal protein kinase domain followed by a conserved calmodulin-binding domain with significant similarity to that of death-associated protein kinase 1 (DAPK1), a positive regulator of programmed cell death.
DDIT3	DNA Damage Inducible Transcript 3. DDIT3, a member of the C/EBP family of transcription factors, functions as a stress-responsive protein, primarily acting as a dominant-negative inhibitor by forming heterodimers with other C/EBP members and preventing their DNA binding, playing a role in adipogenesis, erythropoiesis, and ER stress response.
E2F8	E2F Transcription Factor 8. The encoded protein regulates progression from G1 to S phase by ensuring the nucleus divides at the proper time.
FGF1	Fibroblast Growth Factor 1. This protein functions as a modifier of endothelial cell migration and proliferation, as well as an angiogenic

	factor. It acts as a mitogen for a variety of mesoderm- and neuroectoderm-derived cells in vitro, thus is thought to be involved in organogenesis. Reduction in angiogenesis and impact on tumor microenvironment e.g. tumor-associated fibroblasts.
GINS2	A subunit of the GINS complex, is crucial for DNA replication initiation and progression of replication forks, acting as a core component of the CMG helicase. It's also implicated in cancer development, with overexpression linked to poor prognosis in various cancers. Carcinoma.
HSPA1A	a 70kDa heat shock protein which stabilizes existing proteins against aggregation and mediates the folding of newly translated proteins in the cytosol and in organelles.
HSPA1B	a 70kDa heat shock protein which stabilizes existing proteins against aggregation and mediates the folding of newly translated proteins in the cytosol and in organelles.
HSPA8	Heat Shock 70kDa Protein 8. It functions as a chaperone, and binds to nascent polypeptides to facilitate correct folding. It also functions as an ATPase in the disassembly of clathrin-coated vesicles during transport of membrane components through the cell.
IL1B	Interleukin-1 Beta. This protein is a pro-inflammatory cytokine that plays a crucial role in the immune response. It initiates inflammation in response to infection, injury, or tissue damage. It also promotes the activation of immune cells, including T cells, B cells, and macrophages.
IL1RL1	A member of the interleukin 1 receptor family. Involved in IL-1 family signaling pathways and PI5P, PP2A and IER3 regulated PI3K/AKT signaling.
KIF18A	Kinesin Family Member 18A. KIF18A is a plus-end directed motor protein, and migrates to the plus ends of the spindle during early mitosis. KIF18A has been considered as a cancer target because it is overexpressed in many cancer types and mouse studies suggest it is dispensable in somatic cells.
LFNG	The LFNG gene encodes a glycosyltransferase that modifies Notch receptors, playing a crucial role in embryonic development and Notch signaling, particularly in establishing somite boundaries.
MAP2K6	Mitogen-Activated Protein Kinase Kinase 6. This gene encodes a member of the dual specificity protein kinase family, which functions as a mitogen-activated protein (MAP) kinase kinase. This protein phosphorylates and activates p38 MAP kinase in response to inflammatory cytokines or environmental stress.
MKI67	Marker Of Proliferation Ki-67. Involved in regulation of chromosome segregation and regulation of mitotic nuclear division. Implicated in several diseases, including Crohn's disease; colorectal cancer; endocrine gland cancer (multiple) etc. Ki67 indicates cells engaged in the cell cycle. Beneficial to have target cells no longer proliferating.

MXD3	The encoded protein forms a heterodimer with the cofactor MAX which binds specific E-box DNA motifs in the promoters of target genes and regulates their transcription. Disruption of the MAX-MXD3 complex is associated with uncontrolled cell proliferation and tumorigenesis.
NCAPH	NCAPH stabilizes GEN1 in chromatin and maintain chromosome stability. It is a prognostic biomarker and associated with immune infiltrates in lung adenocarcinoma. It promotes cell proliferation and inhibits cell apoptosis of bladder cancer cells through MEK/ERK signaling pathway.
NLRC4	NLR Family CARD Domain Containing 4. This protein plays essential roles in innate immune response to a wide range of pathogenic organisms, tissue damage and other cellular stresses.
NLRP3	NLR Family Pyrin Domain Containing 3. This protein interacts with the apoptosis-associated speck-like protein PYCARD/ASC, which contains a caspase recruitment domain, and is a member of the NLRP3 inflammasome complex. This complex functions as an upstream activator of NF-kappaB signaling, and it plays a role in the regulation of inflammation, the immune response, and apoptosis.
NLRP10	NLR Family Pyrin Domain Containing 10. This protein likely plays a regulatory role in the innate immune system. The protein belongs to the signal-induced multiprotein complex, the inflammasome, that activates the pro-inflammatory caspases, caspase-1 and caspase-5.
NUF2	NUF2 Component Of NDC80 Kinetochore Complex. The encoded protein is found to be associated with centromeres of mitotic HeLa cells. Diseases associated with NUF2 include Microcephaly and Ovary Serous Adenocarcinoma.
PPP2R5B	Protein Phosphatase 2, Regulatory Subunit B. Protein phosphatase 2A is one of the four major Ser/Thr phosphatases, and it is implicated in the negative control of cell growth and division.
SPC24	This protein is predicted to contribute to GTPase activator activity, and is also involved in NLS-bearing protein import into nucleus.
SPC25	Spindle Pole Body Component 25 Homolog. This gene encodes a protein that is involved in kinetochore-microtubule interaction and spindle checkpoint activity.
TLR3	Toll Like Receptor 3. The protein encoded by this gene is a member of the Toll-like receptor (TLR) family which plays a fundamental role in pathogen recognition and activation of innate immunity.
TOP2A	DNA Topoisomerase II Alpha. An enzyme that controls and alters the topologic states of DNA during transcription. This nuclear enzyme is involved in processes such as chromosome condensation, chromatid separation, and the relief of torsional stress that occurs during DNA transcription and replication.

TP73	Tumor Protein P73. This gene encodes a member of the p53 family of transcription factors involved in cellular responses to stress and development.
TREM1	Triggering Receptor Expressed On Myeloid Cells 1. This protein amplifies neutrophil and monocyte-mediated inflammatory responses triggered by bacterial and fungal infections by stimulating release of pro-inflammatory chemokines and cytokines.
TREM2	Triggering Receptor Expressed On Myeloid Cells 2. This protein functions in immune response and may be involved in chronic inflammation by triggering the production of constitutive inflammatory cytokines.
UGT1A6	This gene encodes a UDP-glucuronosyltransferase, an enzyme of the glucuronidation pathway that transforms small lipophilic molecules, such as steroids, bilirubin, hormones, and drugs, into water-soluble, excretable metabolites.
WNT4	Wnt Family Member 4. This protein has been implicated in oncogenesis and in several developmental processes, including regulation of cell fate and patterning during embryogenesis.
ZNF 488	Zinc Finger Protein 488. This protein predicted to be involved in oligodendrocyte development and regulation of DNA-templated transcription. Predicted to act upstream of or within negative regulation of DNA-templated transcription and positive regulation of oligodendrocyte differentiation.
ZWINT	ZW10 Interacting Kinetochore Protein. This gene encodes a protein that is involved in kinetochore function. It interacts with ZW10, another kinetochore protein, possibly regulating the association between ZW10 and kinetochores.

Supplemental Table 2. RNA primer sets used for the rt-PCR studies.

Name	Forward primer	Reverse primer
ALDH3A1	CCAGCAACGACAAGGTGATTAAG	AGAGAGTGCAAGGTGATGTG
AurKB	GGACACCCGACATCTTAAC	CTTTTCTTCTCCCGAGCCAAG
BIRC5	ACCACCGCATCTCTACATTC	CAAGTCTGGCTCGTTCTCAG
Bub1	CAGGAAAGGTCCGAGGTTAATCC	CACTGGTGTCTGCTGATAG
CDK1	TTTCAGAGCTTTGGGCACTC	ATGCTAGGCTTCCTGGTTTC
CENPA	CCTACAAGAGGCAGCAGAAG	GCACATCCTTTGGGAAGAG
CENPM	CTCGATGCTCAAAGAGGACTG	ACACGATCAGGTCAATTCGGG
CSF2	ATGGCCAGCCACTACAAG	GCAGAAAGTCCTTCAGGTTC
CXCL3	GCCCAAACCGAAGTCATAG	CTTCTCTCCTGTCAGTTGGTG
CXCL8	TTGGCAGCCTTCCTGATTTC	GGTGGAAAGGTTTGGAGTATG

**CXCL10**	**GTCCACGTGTTGAGATCATTG**	**GACCTTTCCTTGCTAACTGC**
**DAPK2**	**TTTGGGACGCCGGAATTTGTTG**	**GATGTAGGTGATGACGCCTATG**
**DDIT3**	**CGAGCTCTGATTGACCGAATG**	**TCTGGGAAAGGTGGGTAGTG**
**E2F8**	**CTGAGTAGCCTGGATCTTATC**	**CTGGAGCCACTGGTATTTG**
**FGF1**	**AGGCTGGAGGAGAACCATTAC**	**CGTTTGCAGCTCCCATTCTTC**
**GINS2**	**GAGGGATCATGAACGAAAGG**	**CTTCGGGATGTTGTCTGAAG**
**HSPA1A**	**GGAGCTTCAAGACTTTGC**	**GCAAGTTCAGTACTTCACC**
**HSPA1B**	**CCATTGAGGAGGTGGATTAG**	**GCAGCAAAGAGCTGAAGCAG**
**HSPA8**	**GATCTTGGCACCACCTACTC**	**CCGTTCAGTGTCCGTAAAG**
**IL1B**	**ACCTCCAGGGACAGGATATG**	**GCCCAAGGCCACAGGTATTTTG**
**IL1RL1**	**AATGGGCTGGCTTGTCTAG**	**CTTACGGTGTGCCTTCTCAAG**
**KIF20A**	**CAGACTGCTCTGTCGTCTCTAC**	**GAAGGTAACAAGGGCCTAACCC**
**LFNG**	**CGCTGAGCTACGGTATGTTTG**	**GTACAGGTGGCAGTGGATG**
**MAP2K6**	**CCACACCACCTCGAGATTTAG**	**CTCGTCCCAGTTCCATTATAG**
**MKI67**	**CTGCCACTAAGCCAGAAAATC**	**AACTTGAGTGAGCCACAGAG**
**MXD3**	**ATGTGGAGAGCCTGGTGTTTG**	**CCTGGGTGAGGAACATCATAG**
**NCAPH**	**ACAACAACCCTAACGACACCTC**	**CCCAACAGGTCCCACAAATAAG**
**NLRC4**	**ATCCAGGTGCTTGAGGAATC**	**GCGTTGTTTGTGTGTGAGAG**
**NLRP3**	**AAGTGGACTGCGAGAAGTTC**	**CGTTCGTCCTTCCTTCCTTTTC**
**NLRP10**	**GACCAGCTCAGCCATATTTG**	**CCTGGTTGTATCTGCCATTG**
**NUF2**	**GAGGTGCTGTCTATGAACGAG**	**TCTCCCTTTCAGCAGCATC**
**PPP2R5B**	**CACACCTGCAGCTGGTATATG**	**CAGGAGCATCAGGACAAAC**
**SPC24**	**CTGGAAGAGCTCAAGGAGATTG**	**GTAAAGTTGAGCCACGTACAC**
**SPC25**	**GGACGAACTGGCACTTTTC**	**ATCTCTTAGTCCCGCCATC**
**TLR3**	**TCCACCACCAGCAATACAAC**	**GTTGTGGAAGCCAAGCAAAG**
**TOP2A**	**TGTGGAAACAGCCAGTAGAG**	**TCTCCATTGAAGGGCTTGAG**
**TP73**	**CTCTCGCAGTATGTGGATGAC**	**GAAGTTGTACAGGATGGTGGTG**
**TREM1**	**TCTCAGAACTCCGAGCTGCAAC**	**GCTGGCAAACTTCTCTAGCGTG**
**TREM2**	**ATGTGGAGCACAGCATCTC**	**AGGAGGAGAAGGATGGAAG**
**UGT1A6**	**ACAGGGACACCCTGAACTTC**	**CACAGATGGTAGGCCCAAATAC**
**WNT4**	**TCAGGCTCCTGTGAGGTAAAG**	**CCTCAGTGGCACCATCAAACTTC**
**ZNF488**	**GCTCCACTCTGTAGCACTTTTC**	**GAAGTGGTGGAGGATGATGAG**

**ZWINT**	**GAGCTTGACAGGGTGTTTC**	**AGCTGGAGGAAGGTCTGATAC**

Supplemental Table 3. A. Geometric properties for the jetted JNPs from ImageJ analyses. **B.** Properties of the suspended JNPs obtained from DLS**.**

A. Geometric properties of the jetted JNPs obtained from ImageJ analysis of the SEM micrograph, n > 5000.

Average (Q1/Med./Q3)	**Diameter (nm)**	**Anisotropy**	**Circularity**	**Roundness**
**JNP-CTR**	354.0 ± 155.4 (243.8/326.1/421.8)	1.26 ± 0.37 (1.07/1.15/1.28)	0.92 ± 0.13 (0.89/0.97/1.00)	0.83 ± 0.15 (0.78/0.87/0.94)
**JNP-4HPR**	321.8 ± 130.1 (230.05/299.6/387.0)	1.22 ± 0.30 (1.06/1.13/1.26)	0.84 ± 0.15 (0.77/0.90/0.95)	0.85 ± 0.13 (0.79/0.89/0.94)
**JNP-4HPR TCZ**	243.9 ± 101.7 (117.4/216.8/281.2)	1.31 ± 0.35 (1.10/1.19/1.39)	0.87 ± 0.13 (0.82/0.91/0.96)	0.80 ± 0.15 (0.72/0.84/0.91)

B. Properties of the suspended JNPs obtained from DLS. Numbers are average of a minimum of 3 samples.

	**Diameter (H_2O)**	**Diameter (dPBS)**	**PDI (H_2O)**	**PDI (dPBS)**	**Zeta potential (H_2O)**	**Zeta potential (dPBS)**
**JNP-CTR**	490.5 ± 47.9 nm	440.3 ± 14.1 nm	0.185 ± 0.052	0.135 ± 0.055	5.6 ± 0.3 mV	-1.3 ± 0.2 mV
**JNP-4HPR**	502.4 ± 74.3 nm	394.1 ± 51.2 nm	0.240 ± 0.05	0.26 ± 0.05	0.5 ± 0.6 mV	-1.9 ± 0.6 mV
**JNP-4HPR TCZ**	463.6 ± 8.3 nm	365.4 ± 27.1 nm	0.180 ± 0.07	0.20 ± 0.07	6.8 ± 3.1 mV	-1.5 ± 0.8 mV
